# Protein Composition and Baking Quality of Wheat Flour as Affected by Split Nitrogen Application

**DOI:** 10.3389/fpls.2019.00642

**Published:** 2019-05-15

**Authors:** Cheng Xue, Andrea Matros, Hans-Peter Mock, Karl-Hermann Mühling

**Affiliations:** ^1^College of Resources and Environmental Sciences, Hebei Agricultural University, Baoding, China; ^2^Faculty of Agricultural and Nutritional Sciences, Institute of Plant Nutrition and Soil Science, Kiel University, Kiel, Germany; ^3^Department of Physiology and Cell Biology, Leibniz Institute of Plant Genetics and Crop Plant Research, Gatersleben, Germany

**Keywords:** wheat, split N application, baking quality, proteomics, gliadin, glutenin

## Abstract

Baking quality of wheat flour is determined by grain protein concentration (GPC) and its composition and is highly influenced by environmental factors such as nitrogen (N) fertilization management. This study investigated the effect of split N application on grain protein composition and baking quality of two winter wheat cultivars, Tobak and JB Asano, belonging to different baking quality classes. Bread loaf volumes in both cultivars were enhanced by split N application. In contrast, GPC was only significantly increased in JB Asano. Comparative 2-DE revealed that the relative volumes of 21 and 28 unique protein spots were significantly changed by split N application in Tobak and JB Asano, respectively. Specifically, the alterations in relative abundance of certain proteins, i.e., globulins, LMW-GS, α-, and γ-gliadins as well as α-amylase/trypsin inhibitors were more sensitive to split N application. Furthermore, certain proteins identified as globulins and alpha-amylase inhibitors were changed in both wheat cultivars under split N application. These results implied that the functions of these unique proteins might have played important roles in affecting baking quality of wheat flour, especially for cultivars (i.e., Tobak in the present study) the baking quality of which is less dependent on GPC.

## Introduction

Grain protein concentration (GPC) is widely used as the main parameter in evaluating baking quality of wheat products and a higher price is usually achieved with high-protein bread wheat. However, the baking quality is complex and is determined not only by GPC but also by its composition (Chaudhary et al., 2016). Based on the solubility in different solvents, the major flour protein types are classified into albumins, globulins, gliadins, and glutenins (Shewry and Halford, 2002). Gluten proteins (gliadins and glutenins) play important roles in determining baking quality of wheat flour as gliadins mainly contribute to dough viscosity and extensibility, while glutenins to dough strength and elasticity (Wieser, 2007).

One well-known effect of fertilization with nitrogen (N) is the increase in GPC. Therefore, to produce high quality wheat and to achieve optimum economic benefits, farmers make strategic decisions about the handling of N fertilization such as N rate, timing and splitting. Fractionation studies demonstrated that additional N increased GPC mainly through the enhanced gliadins and glutenins, as well as the increased proportions of HMW-GS (high-molecular-weight glutenin subunits) and ω-gliadins while decreased proportions of LMW-GS (low-molecular-weight glutenin subunits) (Wieser and Seilmeier, 1998; Xue et al., 2016a,b). Apart from total N fertilization rate, split N application (splitting of the same N fertilizer rate distributed in several applications at different growth stages) enhances N use efficiency thus providing more available N for the plant (Ercoli et al., 2013). Moreover, it has been reported that N fertilizer applied at late growth stages of crops compared to early stages favors protein build-up in the grain over an increased yield (Bogard et al., 2010). Therefore, split N application is supposed to be an effective way in improving baking quality of wheat. Our previous studies using two winter wheat cultivars (Tobak, belonging to quality class B, had similar loaf volume but lower GPC comparing to JB Asano which was classified as class A) clearly separated influences from additional N application and split N application and demonstrated that split N application significantly improved bread loaf volume of both wheat cultivars (Tobak and JB Asano) mainly through the proportionally increased gliadin and glutenin fractions as well as certain HMW-GS as fractionated by sodium dodecyl sulfate polyacrylamide gel electrophoresis (SDS-PAGE) (Xue et al., 2016a,b). However, the GPC was only significantly increased in one cultivar (JB Asano), while remained unchanged in another one (Tobak) as influenced by split N application. These results strongly indicated that split N application caused important changes in N partitioning in grain protein, which were supposed to play key roles in determining baking quality of the resulting wheat flour.

The composition of wheat flour protein is highly complex. However, it is difficult to separate proteins, e.g., LMW-GS and some of the gliadins since they are overlapped in molecular weight (MW) through approaches of SDS-PAGE or chromatographic methods. The use of two-dimensional gel electrophoresis (2-DE) allows better separation of individual proteins and better comparison for subsequent identification of individual proteins. Many studies have used 2-DE to determine influences of environmental factors (e.g., high temperature, drought, or fertilization) on wheat grain protein composition (Dupont et al., 2006; Grove et al., 2009; Zörb et al., 2009; Altenbach et al., 2011; Hurkman et al., 2013; Brzozowski and Stasiewicz, 2017; Wang et al., 2017). However, studies on split N application effect on grain protein composition using 2-DE have not been reported.

In this paper, wheat flours from two N fertilization treatments (with and without split N applied at late boot stage) were selected from our previous study using two cultivars (Xue et al., 2016a). The method of proteomics was conducted to determine the alterations in flour protein composition as well as to identify the specific proteins as influenced by split N application. The objectives of this study were (i) to evaluate the changes in protein composition as influenced by split N application, (ii) trying to identify unique proteins that were more sensitive to split N application which might play crucial roles in affecting baking quality of wheat flour. These results will contribute to better understanding of the improvement of baking quality by split N application.

## Materials and Methods

### Experimental Design

Two winter wheat (*Triticum aestivum* L.) cultivars Tobak (W. von Borries-Eckendorf, Leopoldshöhe, Germany) and JB Asano (Saatzucht Josef Breun, Herzogenaurach, Germany), belonging to different baking quality classes according to the German Federal Office of Plant Varieties, were used in this study. Tobak (class B) had similar loaf volume but lower raw protein concentration comparing to JB Asano (class A).

The N fertilization experiment was conducted in the year of 2011/2012 in Mitscherlich pots (diameter: 21 cm, depth: 21.5 cm) containing 6 kg of soil with supplemental irrigation under natural conditions, i.e., outdoor, except during strong frost and exceptionally high rainfall (monthly precipitation and air temperature were provided in Supplementary Table S1). Nitrogen fertilization treatments N2 and N3 were selected from the previous study (Xue et al., 2016a), and renamed in the present study as follows: (1) early-N treatment, 2 g⋅N⋅pot^-1^ in two doses with 1 g⋅N⋅pot^-1^ applied before seeding and 1 g⋅N⋅pot^-1^ at EC30 (beginning of stem elongation) (Lancashire et al., 1991); (2) split-N treatment, 2 g⋅N⋅pot^-1^ in three doses with 1 g⋅N⋅pot^-1^ applied before seeding, 0.5 g⋅N⋅pot^-1^ at EC30 and 0.5 g⋅N⋅pot^-1^ at EC45 (late boot stage). Other nutrients including P (0.6 g⋅pot^-1^), K (2.3 g⋅pot^-1^), S (0.5 g⋅pot^-1^), Mg (0.33 g⋅pot^-1^), Ca (1.19 g⋅pot^-1^) and the minor elements Cu (10 mg⋅pot^-1^), Zn (15 mg⋅pot^-1^), and Mn (30 mg⋅pot^-1^) were applied before winter wheat seeding. Each treatment was replicated five times. Winter wheat was sown on November 30, 2011 and harvested on August 6, 2012. Nitrogen fertilization was conducted on November 25, 2011, April 2, 2012, and May 30, 2012, respectively. Fungi disease and insects were well controlled by spraying fungicide Capalo three times and insecticide Biscaya once during winter wheat growth.

### Grain Yield, N, and S Concentration

Grain yield (g⋅pot^-1^) was determined as the dry weight of kernels in each pot. Grains were milled in a Titan laminated mill using a 500 μm sieve (Retsch, Haan, Germany). Nitrogen and sulfur (S) concentrations were determined by a CNS elemental analyzer (Flash EA 1112 NCS, Thermo Fisher Scientific, Waltham, MA, United States). Crude protein concentration of wheat flour was calculated by multiplying the N concentration by 5.7.

### Micro Baking Test (MBT)

The MBT with 10 g wholemeal flour of each sample was performed according to Thanhaeuser et al. (2014) and the detailed procedures were described in Xue et al. (2016a). Briefly, the moisture of each sample was measured using infrared moisture analyzer (MA35, Sartorius). The water absorption and dough development time were then determined using a micro-farinograph (Brabender, Duisburg, Germany). Afterward, the MBT was performed and the loaf volumes were measured using Volscan Profiler 600 (Stable Micro Systems, Godalming, United Kingdom).

### Protein Extraction for Two-Dimensional Gel Electrophoresis (2-DE)

The wholemeal flour was further ground to a fine homogeneous powder under liquid N using a mortar. Protein extraction from wholemeal flour for 2-DE was performed using a dithiothreitol (DTT) – trichloroacetic acid (TCA) – acetone precipitation method according to Zörb et al. (2009) with several modifications as described in Xue (2015). Proteins were extracted by adding 1.6 cm^3^ extraction buffer (10% TCA in acetone with 50 mmol⋅dm^-3^ DTT) to 100 mg flour. After vortexing shortly, samples were incubated in an ice-cold ultrasonic bath for 17 min and stored at -20°C overnight. Samples were then centrifuged (16,000 × *g*, 15 min, 4°C) and the supernatant was discarded. The precipitant was resuspended in 1.5 cm^3^ ice-cold dissolving buffer (50 mmol⋅dm^-3^ DTT, 2 mmol⋅dm^-3^ EDTA, in acetone) and incubated in ice-cold ultrasonic bath for 15 min, and then incubated in -20°C for 90 min before centrifugation (16,000 × *g*, 15 min, 4°C). This procedure was repeated and the final pellets were lyophilized under N_2_. To dissolve protein, pellets were resuspended in 1 cm^3^ sample buffer (8 mol⋅dm^-3^ urea, 2 mol⋅dm^-3^ thiourea, 4% CHAPS, 30 mmol⋅dm^-3^ DTT, 20 mmol⋅dm^-3^ Tris base) with 5 mm^3^ protease inhibitor cocktail (Sigma-Aldrich Chemie GmbH, Taufkirchen, Germany), and shaken (2 h, 33°C) and incubated in an ice-cold ultrasonic bath for 15 min before centrifugation (16,000 × *g*, 30 min, 4°C). Finally, supernatant was collected and stored at -20°C for future use.

Protein concentration of each sample was determined using a 2D Quant protein quantification kit (GE Healthcare, Munich, Germany).

### Isoelectric Focusing (IEF) and SDS-PAGE

To separate flour proteins, 2-DE was performed according to O’Farrell (1975) with several modifications as described in Xue (2015). For samples from each biological replicate, two technical replicates were conducted (10 gels for each treatment within each cultivar, 40 gels in total). Commercially purchased immobilized pH gradient (IPG) strips (11 cm, pH 3–10, linear, SERVA, Heidelberg) were used for IEF, which was performed using a Protean IEF Cell system (Bio-Rad). The IPG strips were placed in a tray and 200 mm^3^ protein solution (105 μg protein, 1 mm^3^ protease inhibitor cocktail, 1 mm^3^ IPG buffer pH 3–10, 1 mm^3^ bromophenol blue, adjust to 200 mm^3^ using sample buffer) was applied. Strips were covered with paraffin oil. After isoelectric focusing, strips were covered with paraffin oil and stored at -20°C at least overnight before being used for the second dimension.

The second dimension was performed using medium sized (18 cm × 16 cm × 1.5 mm) SDS-PAGE gels (12.5%). IPG strips were rinsed with running buffer (25 mmol⋅dm^-3^ Tris base, 192 mmol⋅dm^-3^ glycine, 0.1% SDS) and slowly shaken for 20 min in 5 cm^3^ equilibration buffer (50 mmol⋅dm^-3^ Tris–HCl pH 8.8, 6 mol⋅dm^-3^ urea, 30% glycerol, 2% SDS, 1% DTT). The strips were then incubated in 5 cm^3^ equilibration buffer containing 4% iodoacetamide under slow shaking for another 20 min. After that, the strips were rinsed with running buffer again and then mounted onto the gel surface and sealed with 1% agarose containing 0.001% bromophenol blue. A molecular weight standard “Rotimark 10–150” (RotiMark, Roth, Crailsheim, Germany) was added as the marker lane next to the acid side of the IPG strip. Two gels were run simultaneously in the vertical electrophoretic unit (SE600, Hoefer) at 50 mA and 17°C for about 5 h. Gels were then fixed (40% ethanol, 10% acetic acid), stained (one tablet PhastGel^TM^ Blue R in 1.6 dm^3^ 10% acetic acid) and destained (10% acetic acid). Gels were digitized by scanning on an image scanner (Epson Perfection V700) at 300 dpi and 16 bits per pixel.

### 2-DE Gel Densitometric Analysis

Gel images were selected before analysis. For each biological replicate, the gel had a higher resolution within two technical replicates was chosen (Images of 2-DE were provided in Supplementary Figure S1). Computer-assisted 2D analysis of each gel was done using the software Delta 2D 4.0 (Decodon GmbH, Greifswald, Germany). All selected 2D gel images (five replicates for each treatment) of each wheat cultivar were warped using a group warping strategy. Each gel pairs were carefully reviewed and protein spots were matched. Afterward, a fused image (master gel) was created and individual protein spots were detected. The master gel was then used to delete artifacts and specks on individual gels before further processing. Subsequently, spot quantities were normalized by dividing each spot’s volume by the total spot quantity on the image and the protein spots were quantified by their relative intensity on the gel image. The quantity of protein was denoted by % volume.

### Protein Identification

Three independent gels from each treatment in each cultivar were selected for protein identification of selected protein spots which were significantly changed in relative intensity. Protein identification was performed as described in Peukert et al. (2014) with minor modifications. Selected protein spots were manually excised from the 2-DE gels, digested with trypsin, and subjected to mass spectrometry. Acquisition of peptide mass fingerprint data and corresponding LIFT spectra was performed using an ultrafleXtreme MALDI-TOF device (Bruker Daltonics) equipped with a Smartbeam-II laser with a petition rate of 1000 Hz. The spectra were calibrated using external calibration and subsequent internal mass correction. For databank searching, Biotools 3.2 software (Bruker Daltonics) with the implemented MASCOT search engine (Matrix Science) was used, searching against: (a) the non-redundant protein entries in the NCBI data base^1^ (Version 20151012; 72776944 sequences; 26510890717 residues), (b) the unreviewed reference protein data for plants in the UniProt Knowledge Base clustered for 90% homology^2^ (Version 20140128; 1027148 sequences; 364640279 residues), (c) the reviewed reference protein data for plants in the UniProt Knowledge Base clustered for 90% homology [see text footnote 2; SwissProt Version 20140128 (577654 sequences; 214198207 residues)], and (d) the barley genome sequences^3^ (Version 20140128; 79379 sequences; 16296301 residues). Search parameters were as follows: monoisotopic mass accuracy; 50 ppm tolerance; fragment tolerance of 0.3 D; missed cleavages 1; and the allowed variable modifications were oxidation (Met), propionamide (Cys), and carbamidomethyl (Cys). For an accepted annotation, a protein had to be significantly identified from two out of three independent gels. Significance criteria were a minimum score of (a) 78, (b) 85, (c) 70, and (d) 62 at the protein level, and a minimum score of (a) 51, (b) 55, (c) 41, and (d) 30 on the peptide level, for the respective data bases (detailed protein identification data of protein spots were provided in Supplementary Table S2).

### Statistical Analysis

Data for grain yield, protein concentration, N/S ratio, water absorption, development time, stability and loaf volume were presented as the mean value ± standard error of five biological replicates. Differences between treatments and cultivars were separated using two-way analysis of variance, performed with SPSS version 13.0 (Chicago, IL, United States). Variations between treatments and cultivars were checked with the Tukey test.

For the comparisons of the normalized volumes of protein spots, the Student’s *t*-test was performed using the Delta 2D software (Decodon GmbH, Greifswald, Germany) at a significance level of *p* ≤ 0.05.

## Results

### Grain Yield, Protein Concentration, and N/S Ratio

Grain yield of both winter wheat cultivars were not affected by split N application (split-N vs. early-N treatment), ranging from 75 to 80 g⋅pot^-1^ (Table 1). The GPC was significantly influenced by N treatment (Table 1). Split N application increased GPC by 9.1% in JB Asano compared with early-N treatment. Moreover, the ratio of N and sulfur (S) concentration in wheat grain may give an overview of S status of wheat grain. The N/S ratio in both wheat cultivars ranged between 11.0 and 11.8 (below 17), which indicated no S deficiency in the present study. Cultivar differences as well as treatment by cultivar interaction were not detected in grain yield, GPC, or N/S ratio.

**Table 1:** Grain yield, protein concentration, N/S ratio as affected by split N application.

Treatment		Grain yield	Protein concentration	N/S ratio
		(g⋅pot^-1^)	(mg g^-1^ flour)	
Tobak	Early-N	74.5 ± 1.7^b^	88.4 ± 0.8^b^	11.8 ± 0.4^a^
	Split-N	75.5 ± 1.1a^b^	91.9 ± 2.4a^b^	11.1 ± 0.4^a^
JB Asano	Early-N	79.5 ± 2.2^a^	87.0 ± 1.1^b^	11.0 ± 0.6^a^
	Split-N	75.0 ± 1.0a^b^	94.9 ± 1.2^a^	11.8 ± 0.7^a^
*F*-test	Treatment	ns	^∗∗^	ns
	Cultivar	ns	ns	ns
	T × C	ns	ns	ns


*Different letters in the same column represent significant differences of mean values ± standard error of five independent pot replicates at *p* ≤ 0.05 level (Tukey test). T, treatment; C, cultivar; ns, not significant; ^∗^*p* ≤ 0.05; ^∗∗^*p* ≤ 0.01; ^∗∗∗^*p* ≤ 0.001.*

### Flour Water Absorption, Dough Development Time, Stability, and Bread Loaf Volume

Farinograph and micro-scale baking test were conducted to evaluate changes in bread-making quality of wheat flour caused by split N application (Table 2). The water absorption of flour was significantly increased by 2.5 and 4.3% in Tobak and JB Asano by split N application, respectively. Besides, Tobak showed significantly higher (6.5% on average) water absorption than JB Asano. However, no treatment differences were detected in development time and stability of dough from both wheat cultivars as affected by split N application. Again, strong cultivar differences existed in dough development time, being 22.9% longer in JB Asano than Tobak. Despite the inconsistent effects on GPC (Table 1), the bread loaf volumes in both cultivars were significantly improved by split N application, being increased by 5.4 and 10.5% in Tobak and JB Asano, respectively. Besides, cultivar differences existed that Tobak showed higher (3.9% on average) loaf volume compared to JB Asano. The results of GPC and bread loaf volume indicated that the changes in grain protein composition might have exerted great influence on baking quality of wheat.

**Table 2:** Flour water absorption, development time, stability, and bread loaf volume as affected by split N application.

Treatment		Water absorption	Development	Stability	Loaf volume
		(%)	time (min)	(min)	(cm^3^)
Tobak	Early-N	66.8 ± 0.2^b^	4.3 ± 0.2^b^	2.5 ± 0.2^a^	31.4 ± 0.2^b^
	Split-N	68.5 ± 0.1^a^	4.0 ± 0.3^b^	2.6 ± 0.2^a^	33.1 ± 0.3^a^
JB Asano	Early-N	62.2 ± 0.2d	5.3 ± 0.2^a^	2.8 ± 0.2^a^	29.5 ± 0.7c
	Split-N	64.9 ± 0.2c	4.9 ± 0.1^a^	2.5 ± 0.1^a^	32.6 ± 0.3a^b^
*F*-test	Treatment	^∗∗∗^	ns	ns	^∗∗∗^
	Cultivar	^∗∗∗^	^∗∗∗^	ns	^∗^
	T × C	^∗^	ns	ns	ns


*Different letters in the same column represent significant differences of mean values ± standard error of five independent pot replicates at *p* ≤ 0.05 level (Tukey test). T, treatment; C, cultivar; ns, not significant; ^∗^*p* ≤ 0.05; ^∗∗^*p* ≤ 0.01; ^∗∗∗^*p* ≤ 0.001.*

### Grain Protein Profile and Proteome Analyses

**FIGURE 1:**
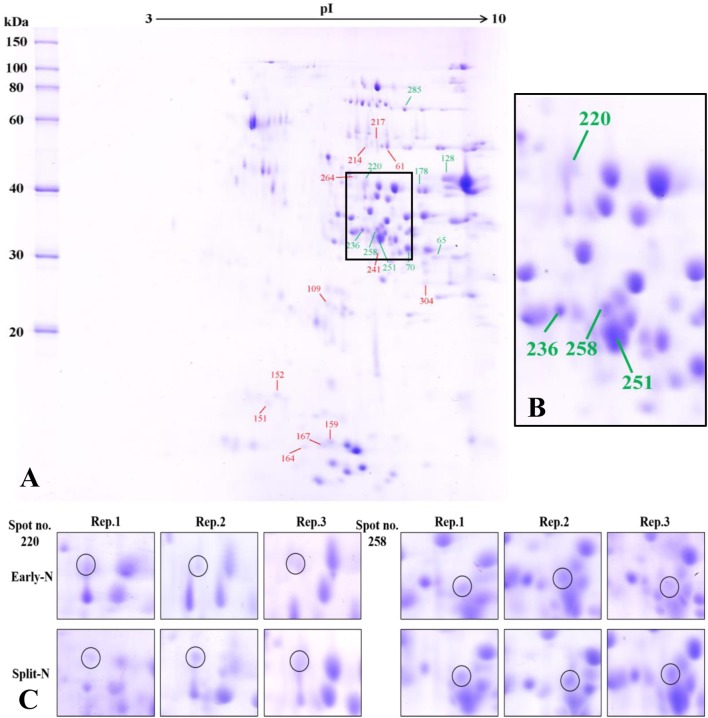
**(A)** Effect of split N application on grain proteome in Tobak. The green and red lines with numbers indicate the spots that were significantly increased or decreased by split N application, respectively. pI, isoelectric point. **(B)** Section part of the panel **(A)** with protein spots that the abundances were changed by more than onefold with split N application. **(C)** Detailed images of protein spots whose abundances were increased by more than 1.5-fold with/without split N application from raw images of three biological replicates, respectively.

**FIGURE 2:**
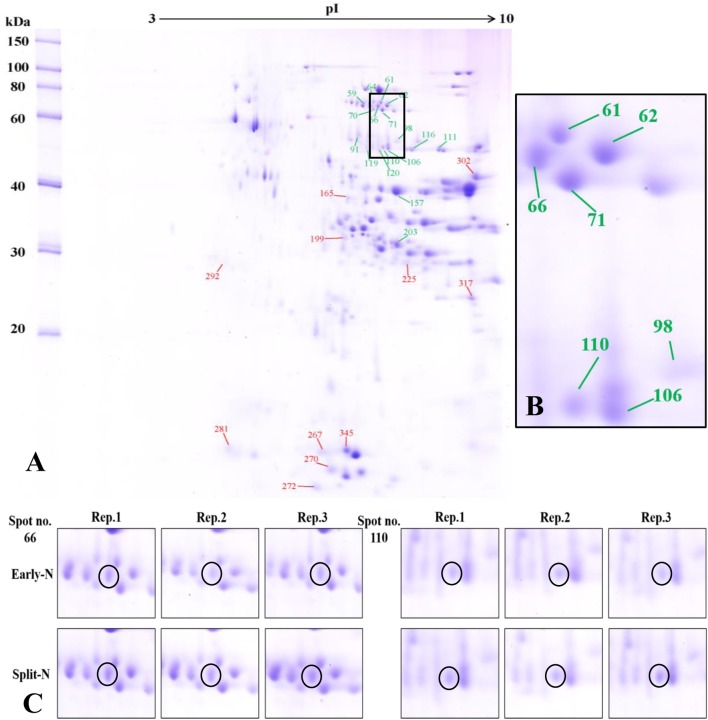
**(A)** Effect of split N application on grain proteome in JB Asano. The green and red lines with numbers indicate the spots that were significantly increased or decreased by split N application, respectively. pI, isoelectric point. **(B)** Section part of the panel **(A)** with protein spots that were increased with split N application. **(C)** Detailed images of protein spots whose abundances were increased by more than 0.5-fold with/without split N application from raw images of three biological replicates, respectively.

The proteome analysis using 2-DE provided insights to the relative changes in abundance of single proteins and to identify the changed proteins. Generally, protein profiles were similar between Tobak and JB Asano. Only quantitative changes in spot volume were detected, whereas no absence of existing protein spots or presence of new protein spots occurred as influenced by split N application. Therefore, the 2-DE protein profiles only for the split-N treatment were depicted for Tobak (Figure 1A) and JB Asano (Figure 2A).

**FIGURE 3:**
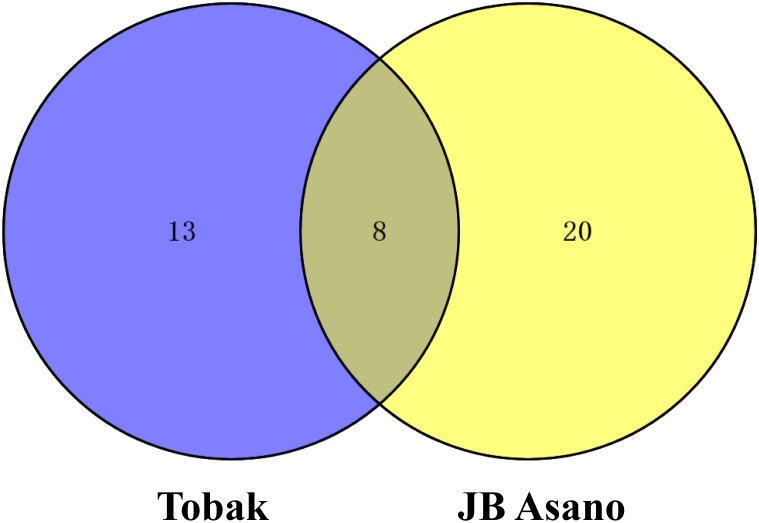
Venn diagram representing the numbers of changed grain protein spots in Tobak and JB Asano with split N application.

**FIGURE 4:**
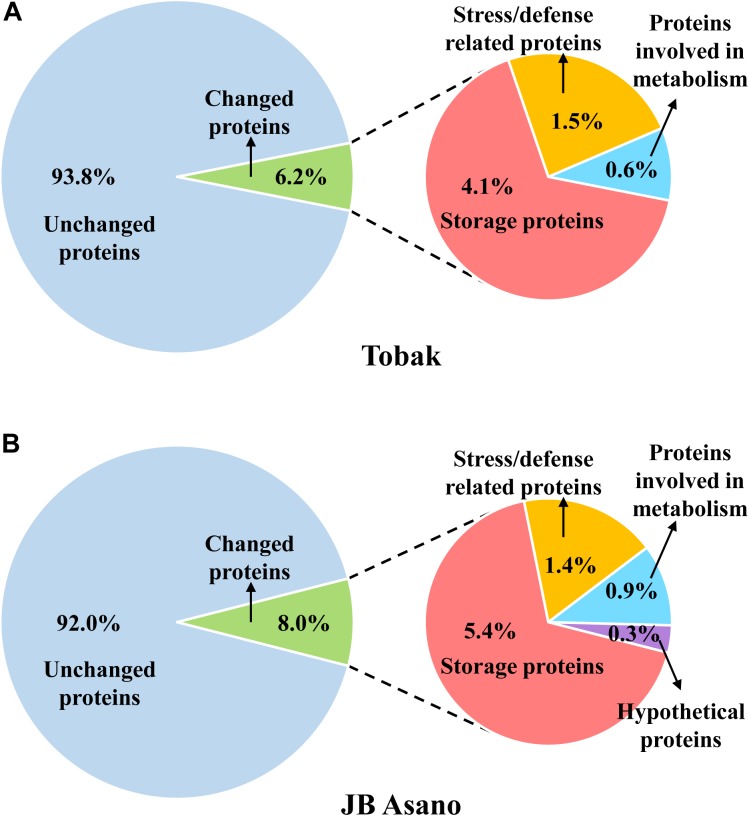
Distribution of the proteins detected in wheat grain and the proteins specifically changed by split N application as well as their functional classification in Tobak **(A)** and JB Asano **(B)**. The percentages of individual protein groups were calculated based on number of protein spots.

In Tobak, a total of 341 protein spots were determined. The average spot volume of 21 protein spots was significantly altered by split N application compared with early-N treatment (Table 3 and Figure 3, 4A), accounting for 6.2% of the total protein spots and for 8.4 and 9.5% of the total spot volumes in early-N and split-N treatments, respectively. Besides, within these 21 spots, the volumes of 9 protein spots were increased, while 12 protein spots were decreased by split N application. In JB Asano, 352 protein spots were detected. The average spot volume of 28 protein spots (17 spots being increased and 11 spots being decreased in relative abundances) was significantly changed by split N application (Table 4 and Figure 3, 4B), accounting for 8.0% of the total protein spots. These significantly altered protein spots taken up 14.7 and 14.6% of the total spot volumes in early-N and split-N treatments, respectively. In terms of cultivar responses, although less protein spots were significantly altered in Tobak (21) than in JB Asano (28), the magnitude of changes in spot volumes was higher in Tobak (4 protein spots changed by more than 100%, 2 protein spots changed by over 150%, Table 3 and Figure 1B,C) than in JB Asano (no protein spots changed by more than 100%, only protein spots no. 66 and 110 were increased by over 50%, Table 4) by split N application.

**Table 3:** Unique protein spots that changed significantly (*p* ≤ 0.05) by split N application in Tobak.

Spot no.^a^	Identification	Accession number	MW (kDa)	pI	Early-N	Split-N	% change (split-N/early-N)
						
					Spot volume (%)^∗^		
285	Globulin-3	B7U6L4^c^	66.3	7.78	0.37 ± 0.04^e^	0.50 ± 0.03	35
214	Globulin-3	B7U6L4^c^	66.3	7.78	0.27 ± 0.01	0.18 ± 0.01	-33
152	Globulin-3	B7U6L4^c^	66.3	7.78	0.26 ± 0.03	0.17 ± 0.03	-35
151	Globulin-3	B7U6L4^c^	66.3	7.78	0.19 ± 0.01	0.13 ± 0.02	-32
61	Globulin-3	B7U6L4^c^	66.3	7.78	0.71 ± 0.07	0.55 ± 0.02	-23
264	Globulin-1	M7ZK46^c^	63.8	6.62	0.27 ± 0.02	0.11 ± 0.03	-59
217	Globulin-1	M8B8C6^c^	51.6	6.81	0.31 ± 0.02	0.22 ± 0.02	-29
178	LMW-GS 5 type III	gi| 17425192^b^	39.8	8.48	0.36 ± 0.03	0.64 ± 0.07	78
128	LMW-GS 1–50	A9UID2^c^	35.1	9.25	1.03 ± 0.10	1.94 ± 0.26	88
258	Alpha-gliadin	gi| 807780722^b^	33.1	8.57	0.16 ± 0.05	0.40 ± 0.06	150
70	Alpha-gliadin	gi| 513129900^b^	32.8	8.24	1.10 ± 0.08	1.48 ± 0.12	35
65	Gamma-gliadin	B8XU42^d^	32.5	7.56	0.18 ± 0.01	0.30 ± 0.04	67
304	Xylanase inhibitor protein I	gi| 20804336^b^	33.2	8.66	0.24 ± 0.03	0.18 ± 0.01	-25
236	Aldose reductase	gi| 474048335^b^	35.9	6.87	0.18 ± 0.06	0.43 ± 0.05	139
220	Glyceraldehyde-3-phosphate dehydrogenase	M7ZRJ1^c^	42.5	9.17	0.05 ± 0.01	0.20 ± 0.01	300
251	Avenin-like b4	A5A4L5^d^	32.7	8.29	0.42 ± 0.06	0.85 ± 0.12	102
241	Avenin-like protein	gi| 558550235^b^	32.4	8.09	0.90 ± 0.18	0.36 ± 0.01	-60
109	1-Cys peroxiredoxin PER1	gi| 75324900^b^	24.0	6.08	0.36 ± 0.04	0.25 ± 0.03	-31
167	Alpha-amylase/trypsin inhibitor CM3	P17314^d^	18.2	7.44	0.19 ± 0.03	0.12 ± 0.01	-37
164	Alpha-amylase/trypsin inhibitor CM2	D2TGC2^d^	15.4	6.86	0.32 ± 0.02	0.14 ± 0.02	-56
159	Dimeric alpha-amylase inhibitor	gi| 65993781^b^	15.1	5.58	0.73 ± 0.06	0.46 ± 0.05	-37


*^*a*^Spot numbers are assigned according to Figure 1A; ^*b*^Data base: NCBI; ^*c*^Data base: UniProt; ^*d*^Date base: SwissProt; ^*e*^Mean values ± standard error of five independent pot replicates; ^∗^the spot volume of each protein spot was significantly changed by split-N treatment compared with early-N treatment at *p* ≤ 0.05 level determined by Student’s *t*-test. MW, molecular weight (theoretical); pI, isoelectric point (theoretical).*

**Table 4:** Unique protein spots that changed significantly (*p* ≤ 0.05) by split N application in JB Asano.

Spot no.^a^	Identification	Accession number	MW (kDa)	pI	Early-N	Split-N	% change (split-N/early-N)
						
					Spot volume (%)^∗^		
70	Globulin-3	B7U6L4^c^	66.3	7.78	0.13 ± 0.01^e^	0.18 ± 0.01	38
61	Globulin-3	B7U6L4^c^	66.3	7.78	0.24 ± 0.01	0.34 ± 0.02	42
64	Globulin-3	B7U6L4^c^	66.3	7.78	0.27 ± 0.01	0.37 ± 0.04	37
66	Globulin-3	B7U6L4^c^	66.3	7.78	0.21 ± 0.01	0.32 ± 0.02	52
62	Globulin-3	B7U6L4^c^	66.3	7.78	0.38 ± 0.03	0.55 ± 0.02	45
71	Globulin-3	B7U6L4^c^	66.3	7.78	0.26 ± 0.02	0.36 ± 0.02	38
106	Globulin-3	B7U6L4^c^	66.3	7.78	0.51 ± 0.04	0.66 ± 0.03	29
116	Globulin-3	B7U6L4^c^	66.3	7.78	0.38 ± 0.02	0.45 ± 0.02	18
111	Globulin-3	B7U6L4^c^	66.3	7.78	0.25 ± 0.03	0.34 ± 0.01	36
110	Globulin-3B	B7U6L5^c^	56.9	7.36	0.16 ± 0.01	0.24 ± 0.01	50
59	Globulin-1 S allele	M8A380^c^	56.9	9.1	0.44 ± 0.02	0.61 ± 0.06	39
91	Globulin-1 S allele	M7ZQM3^c^	55.3	7.77	0.53 ± 0.02	0.64 ± 0.04	21
98	Globulin-1 S allele	M7ZQM3^c^	55.3	7.77	0.20 ± 0.01	0.25 ± 0.02	25
119	Globulin-1 S allele	M8B8C6^c^	51.6	6.81	0.17 ± 0.01	0.22 ± 0.01	29
120	Globulin-1 S allele	gi| 475542024^b^	51.6	6.81	0.22 ± 0.02	0.30 ± 0.03	36
302	S-type LMW-GS L4-292	Q6J162^c^	28.9	8.52	1.75 ± 0.12	1.28 ± 0.12	-27
165	G3P dehydrogenase 1	P26517^d^	36.5	6.67	0.28 ± 0.02	0.20 ± 0.02	-29
225	Avenin-like b1	Q2A783^d^	32.7	8.08	0.18 ± 0.02	0.12 ± 0.01	-33
203	Putative avenin-like b precursor	gi| 89143130^b^	32.3	7.83	0.86 ± 0.05	1.10 ± 0.06	28
199	Glucose and ribitol dehydrogenase-like protein	gi| 475619363^b^	31.6	6.54	0.38 ± 0.03	0.28 ± 0.03	-26
317	Basic endochitinase C	Q9FRV0^d^	28.3	8.82	0.33 ± 0.04	0.07 ± 0.01	-79
157	Gamma prolamin	H8Y0K9^c^	23.2	8.15	2.18 ± 0.11	2.6 ± 0.10	19
292	hypothetical protein F775_14150 (*Aegilops tauschii*)	gi| 475596183^b^	19.9	5.2	0.10 ± 0.01	0.06 ± 0.01	-40
345	Alpha-amylase/trypsin inhibitor CM3	P17314^d^	18.2	7.44	1.68 ± 0.04	1.26 ± 0.10	-25
272	Alpha-amylase inhibitor 0.28	P01083^d^	16.8	7.45	0.74 ± 0.03	0.47 ± 0.07	-36
281	Alpha-amylase/trypsin inhibitor CM16	P16159^d^	15.8	5.31	0.33 ± 0.05	0.16 ± 0.03	-52
270	Alpha-amylase/trypsin inhibitor CM2	P16851^d^	15.4	6.86	0.86 ± 0.06	0.58 ± 0.07	-33
267	Alpha-amylase inhibitor 0.19	P01085^d^	13.3	6.66	0.69 ± 0.02	0.62 ± 0.02	-10


*^*a*^Spot numbers are assigned according to Figure 2; ^*b*^Data base: NCBI; ^*c*^Data base: UniProt; ^*d*^Date base: SwissProt; ^*e*^Mean values ± standard error of five independent pot replicates; ^∗^the spot volume of each protein spot was significantly changed by split-N treatment compared with early-N treatment at *p* ≤ 0.05 level determined by Student’s *t*-test. MW, molecular weight (theoretical); pI, isoelectric point (theoretical).*

These significantly changed protein spots might contribute to the alterations in baking quality of the corresponding wheat flour. Therefore, to better understand their functions in plant metabolism and baking quality, identification of these specific protein spots was conducted. Among the spots that increased with split N application were one spot identified as globulins, two as LMW-GS, two as α-gliadins, one as γ-gliadins as well as three as other proteins, i.e., aldose reductase, glyceraldehyde-3-phosphate dehydrogenase (GAPDH) and avenin-like b4 in Tobak (Table 3), while 15 spots were identified as globulins, one as putative avenin-like b precursor and one as γ-prolamins in JB Asano (Table 4), respectively. Moreover, among the spots that decreased with split N application were six spots identified as globulins, one as xylanase inhibitor, one as avenin-like protein, one as 1-Cys peroxiredoxin PER 1, two as α-amylase/trypsin inhibitors and one as dimeric α-amylase inhibitor in Tobak (Table 3), while one spot was identified as S-type LMW-GS, one as GAPDH, one avenin-like b1, one as glucose and ribitol dehydrogenase-like protein, one as basic endochitinase, one as hypothetical protein, three as α-amylase/trypsin inhibitors and two as α-amylase inhibitors (Table 4), respectively. However, the changes of different protein groups varied between Tobak and JB Asano. In Tobak, among the seven globulin spots changed by split N application, six of them were reduced in volume while all of the 15 globulin spots were increased in volume in JB Asano. Besides, two LMW-GS in Tobak were enhanced in volume while one LMW-GS in JB Asano was decreased in volume. All of the protein spots belonging to α-amylase group were decreased in volume in both Tobak and JB Asano.

## Discussion

Grain protein concentration is widely used as the main criterion in predicting baking quality of wheat flour. However, it was clearly shown that the variation in GPC did not correlate well with baking quality (loaf volume) as affected by split N application in Tobak as well as between cultivars (Tables 1, 2). In fact, we already demonstrated that GPC was not suitable in predicting baking quality of wheat flour as affected by split N application and late N fertilization in these cultivars from pot and field studies (Xue et al., 2016a,b). In terms of cultivar differences, although the bread loaf volume of both cultivars was significantly enhanced by split N application, GPC was only increased in JB Asano. Furthermore, the bread loaf volume of Tobak was 6.4 and 1.5% higher than JB Asano under early-N and split-N treatments, respectively. However, the enhancement of bread loaf volume in Tobak was 5.4%, while in JB Asano, it was 10.5% resulting from split N application compared to early-N treatment (Table 2), which was in agreement with other studies (Gabriel et al., 2017). Based on quality parameters from German Federal Office of Plant Varieties, Tobak is a cultivar that normally contains relatively lower GPC at optimum baking quality as compared to JB Asano. It could be assumed that Tobak might be less dependent on quantity of grain protein to obtain a certain level of baking quality as compared to JB Asano which might need higher GPC to reach similar baking quality. Therefore, these findings highly implied that the alteration in protein composition caused by split N application might have played key roles in determining baking quality of wheat flour, especially for Tobak with less changes (4.0%) compared to JB Asano (9.1%) in GPC by split N application.

The resolution and distribution of the protein profiles in the present study (Figure 1A, 2A) were comparable to other studies using 2-DE to investigate responses of grain protein composition to various environmental conditions (Dupont et al., 2006; Grove et al., 2009; Zörb et al., 2009; Altenbach et al., 2011; Hurkman et al., 2013; Vensel et al., 2014). In this study, the volumes of mainly α-amylase/protease inhibitors and trypsin inhibitors located at the bottom of 2-DE gels with MW ranging from 13.3 to 18.2 kDa were decreased by split N application compared with early-N treatment in both Tobak and JB Asano (Figure 1A, 2A and Tables 3, 4). These findings were in accordance with other studies reporting that the abundances of α-amylase/protease inhibitors and trypsin inhibitors (contain relatively high content of S-rich amino acids, i.e., cysteine and methionine) decreased with post-anthesis fertilization or increased with S fertilization (Grove et al., 2009; Altenbach et al., 2011). These proteins were believed to be important in the defense mechanisms of the seed against insects and microbial pests while also to be the allergens associated with baker’s asthma for human (Feng et al., 1996). The reason for changes of these proteins may be attributed to the inter-dependence existing in plant N and S metabolism, e.g., the content of S-containing amino acids was as high as 12.3% for an α-amylase inhibitor sequence while was only 0–0.6% for most ω-gliadins (Wieser and Seilmeier, 1998; Zhao et al., 1999; Daniel and Triboi, 2000; Wieser et al., 2004; Altenbach et al., 2011; Zhong et al., 2019). Therefore, although the N/S ratio (Table 1) indicated that there was no apparent S deficiency in the present study (Randall et al., 1981), it seemed the changes in different protein classes as affected by split N application were related to the proportions of S-containing amino acids in the proteins from these classes. Besides, the proteins of the α-amylase/trypsin inhibitor family were considered as allergens, especially for the Baker’s asthma (Houba et al., 1998). Therefore, the reduction in abundance of these proteins with split N application may potentially alleviate the risk of the allergic disease.

The most abundant protein subunits in wheat grains were α-, γ-gliadins and LMW-GS, distributed mainly between 30 and 50 kDa in the protein profiles (Figure 1A, 2A). Within these protein spots, two LMW-GS, two α-gliadins and one γ-gliadin were increased in Tobak while one LMW-GS was decreased in JB Asano as affected by split N application, respectively (Tables 3, 4). Baking quality of wheat flour is highly dependent on the quantity and composition of gluten proteins. However, the changes in protein and baking quality might result from different aspects between these two cultivars. Generally, with the enhancement in GPC, the relative proportions of ω-gliadins and HMW-GS increase, while that of α-, γ-gliadins and LMW-GS decrease (Wieser and Seilmeier, 1998; Altenbach et al., 2011). However, no significant increases of ω-gliadins and HMW-GS in both cultivars while only a decrease of one LMW-GS in JB Asano by split N application were detected in the present study. Therefore, in association of these results with changes of GPC and loaf volume in JB Asano, the improvement in baking quality of JB Asano might mainly be attributed to the significantly increased GPC (Tables 1, 2, 4). However, for Tobak, the improvement in baking quality was mainly due to the alterations in protein composition without significant changes in GPC. As described above, although less protein spots were changed by split N application in Tobak, the changes were to a higher magnitude comparing to that in JB Asano (Tables 3, 4). Generally, the improvement of bread-making or baking quality of wheat flour resulted mainly from the increases in HMW-GS while decreases in LMW-GS (Garrido-Lestache et al., 2004; Fuertes-Mendizábal et al., 2010; Zörb et al., 2010). Furthermore, our previously published results using SDS-PAGE agreed well with these viewpoints (Xue et al., 2016a,b). However, although no significant changes in HMW-GS were detected in Tobak from 2-DE, the increases in the abundances of LMW-GS, α- and γ-gliadins caused by split N application might exert positive influences on baking quality of flour, especially LMW-GS (spot no. 128) and α-gliadins (spot no. 70) which individually accounted for more than 1% of the total grain proteins detected in the present study.

Globulin proteins are located mainly in the aleurone layer and embryo. Increases in the abundance of globulins have been reported normally under stress, e.g., high temperatures (Hurkman et al., 2009; Majoul-Haddad et al., 2013). Besides, it has been reported that the abundance of globulins was influenced by fertilization management as well (Altenbach et al., 2009). In the present study, globulins responded differently to split N application between Tobak and JB Asano (Tables 3, 4). All the 15 significantly changed globulins in JB Asano were increased while most of which in Tobak was decreased by split N application. The increases in globulins abundance may reduce the nutritional quality of wheat flour since globulin-3 has been associated with the incidence of diabetes (Koziol et al., 2012). Moreover, wheat globulins are also likely to be glycosylated which may result in improved emulsifying capacity and emulsion stability (Altenbach et al., 2009). From this aspect, the enhanced abundance of globulins may exert improvement in some of the functional properties of dough in JB Asano. However, for Tobak, the reduction of globulins abundances might imply that more of other proteins (i.e., gliadins and glutenins) were enhanced by split N application since the GPC was not significantly changed.

## Conclusion

Split N application significantly improved baking quality of wheat flour. Grain protein concentration and composition of different cultivars responded differently to split N application. The alterations in relative abundance of certain proteins, i.e., globulins, LMW-GS, α- and γ-gliadins as well as α-amylase/trypsin inhibitors were more sensitive to split N application, which implied that the functions of these unique proteins may have played crucial roles in affecting baking quality of wheat flour, especially for cultivars the baking quality of which is less dependent on grain protein concentration.

## Data Availability

All datasets for this study are included in the manuscript and the Supplementary Files.

## Author Contributions

K-HM conceived and planned the study. CX conducted the experiments and analyses with supervision of K-HM. AM and H-PM conducted the protein identification of gel spots. CX wrote the manuscript. H-PM and K-HM revised and commented on the manuscript.

## Conflict of Interest Statement

The authors declare that the research was conducted in the absence of any commercial or financial relationships that could be construed as a potential conflict of interest.

## Footnotes

1 http://www.ncbi.nlm.nih.gov/protein

2 http://www.uniprot.org/uniprot/

3 http://webblast.ipk-gatersleben.de/barley/

## Supplementary Material

Table S1 Monthly precipitation and temperature during experimental year and the past 30 years.

Table S2 Detailed information of protein identification of Tobak and JB Asano.
