# Supplementary material for: Protein Composition and Baking Quality of Wheat Flour as Affected by Split Nitrogen Application
Source: Front Plant Sci. 2019 May 15;10:642. doi: 10.3389/fpls.2019.00642 (PMC6530357; doi:10.3389/fpls.2019.00642)
Supplement: Supplementary file 3 [file Image_1.pdf]

Supplementary Figure S1 2-DE gel images of Tobak and JB Asano.

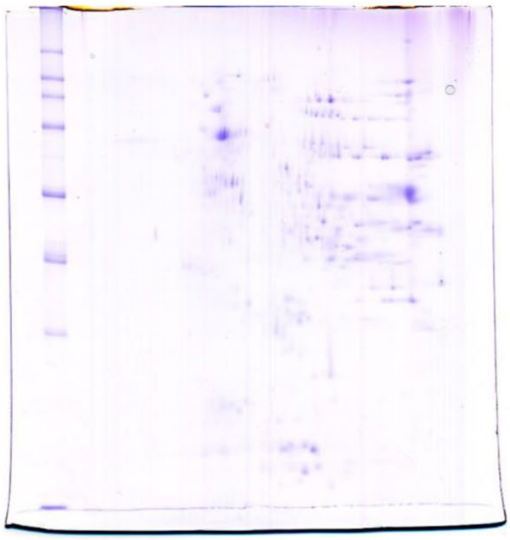

Tobak-Early N treatment-
Replicate 1

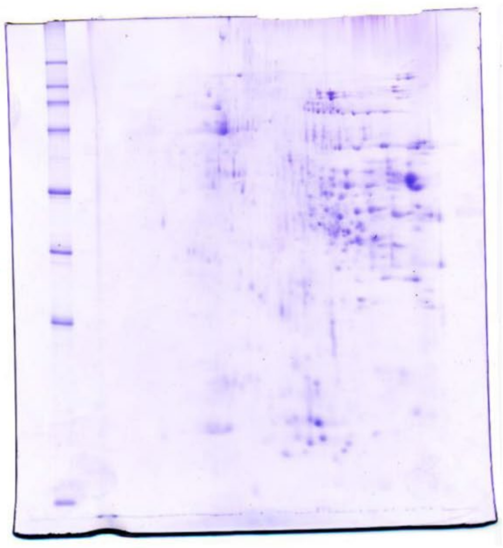

Tobak-Split N treatment-
Replicate 1

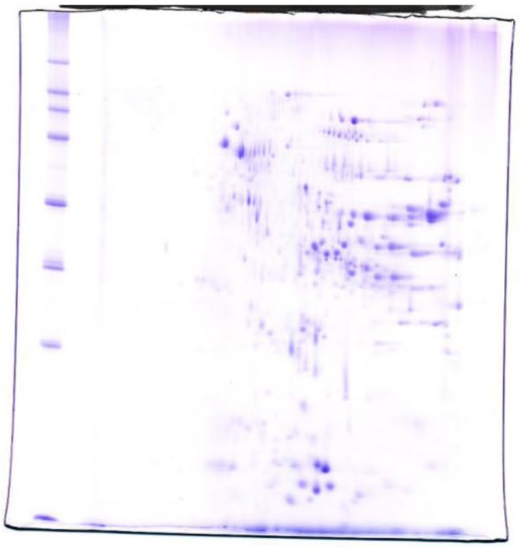

JB Asano-Early N treatment-
Replicate 1

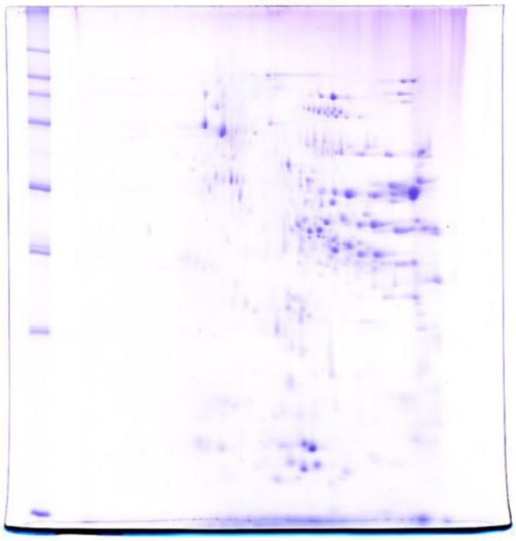

JB Asano-Split N treatment-
Replicate 1

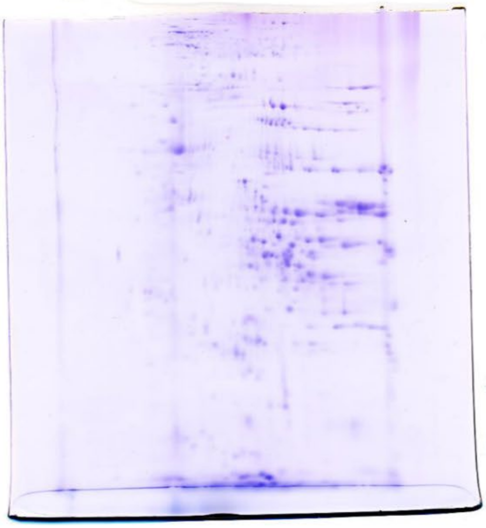

Tobak-Early N treatment-
Replicate 2

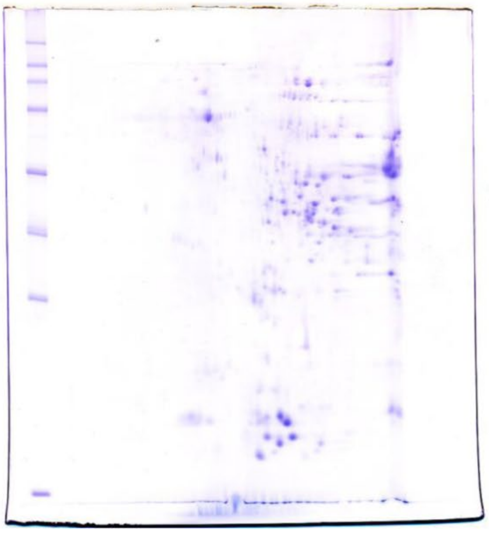

Tobak-Split N treatment-
Replicate 2

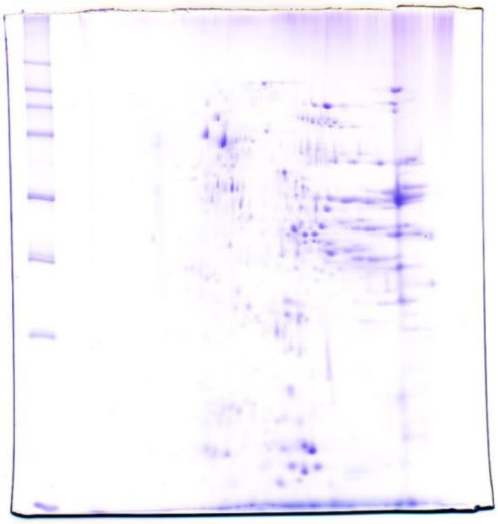

JB Asano-Early N treatment-
Replicate 2

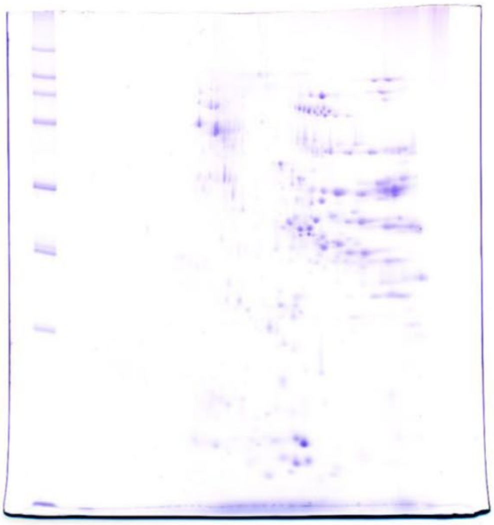

JB Asano-Split N treatment-
Replicate 2

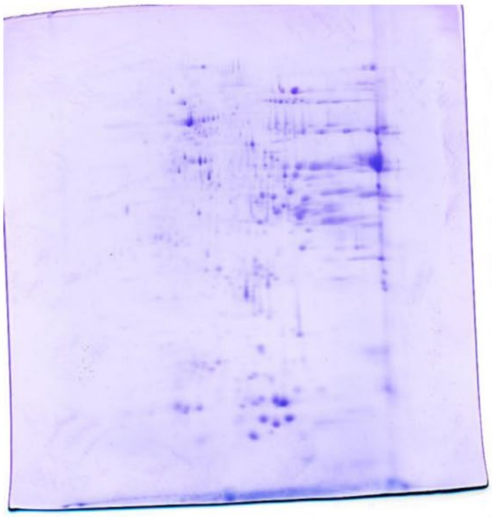

Tobak-Early N treatment-
Replicate 3

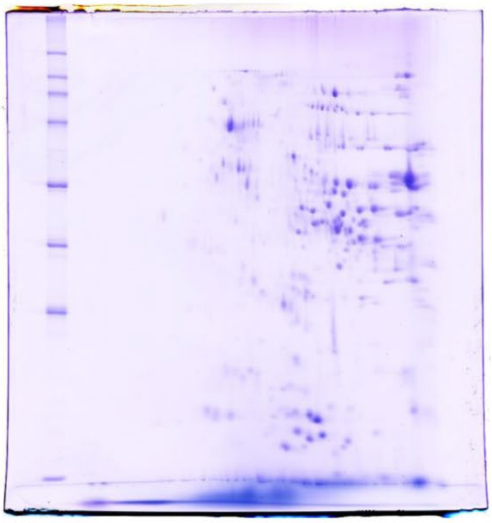

Tobak-Split N treatment-
Replicate 3

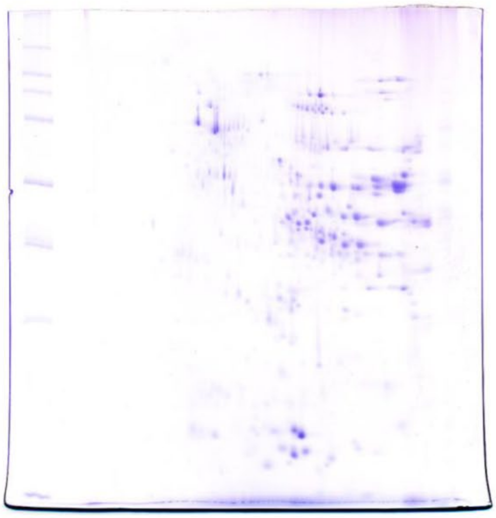

JB Asano-Early N treatment-
Replicate 3

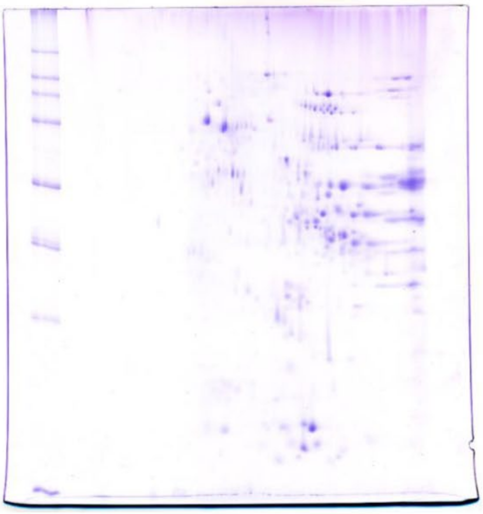

JB Asano-Split N treatment-
Replicate 3

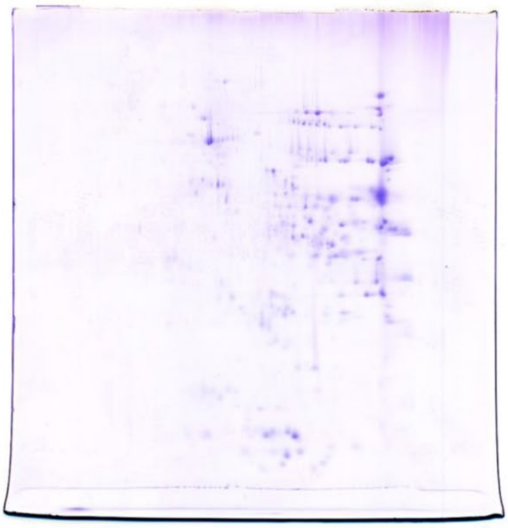

Tobak-Early N treatment-
Replicate 4

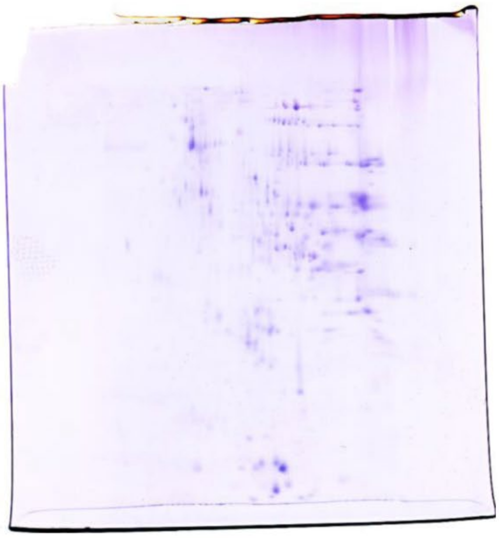

Tobak-Split N treatment-
Replicate 4

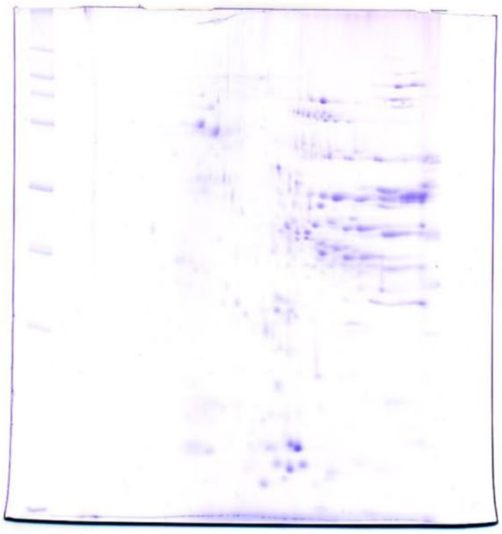

JB Asano-Early N treatment-
Replicate 4

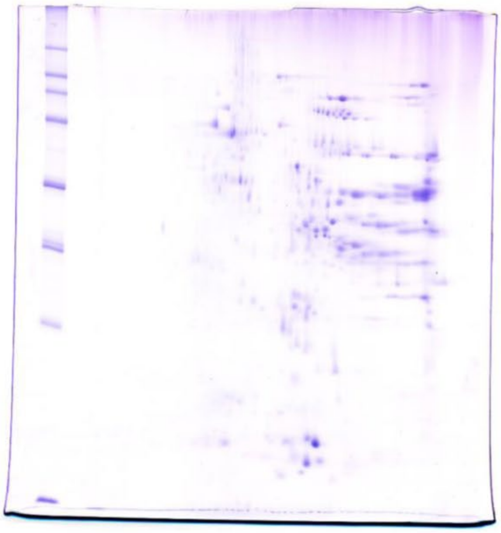

JB Asano-Split N treatment-
Replicate 4

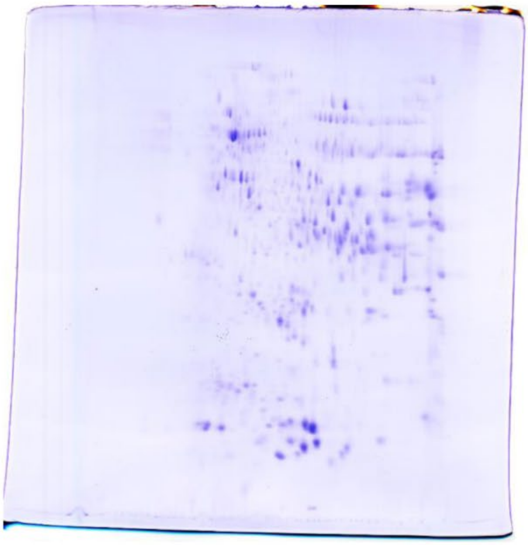

Tobak-Early N treatment-
Replicate 5

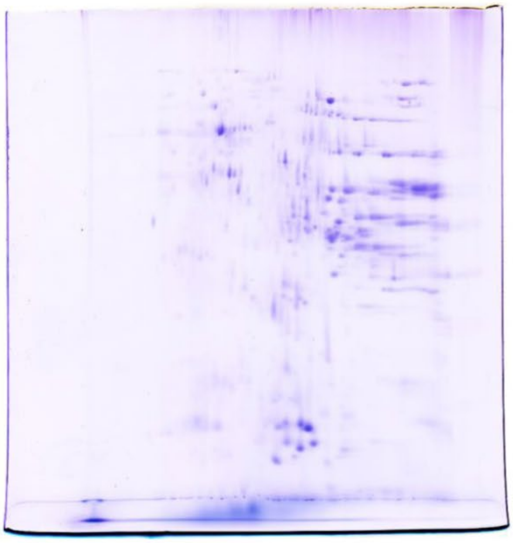

Tobak-Split N treatment-
Replicate 5

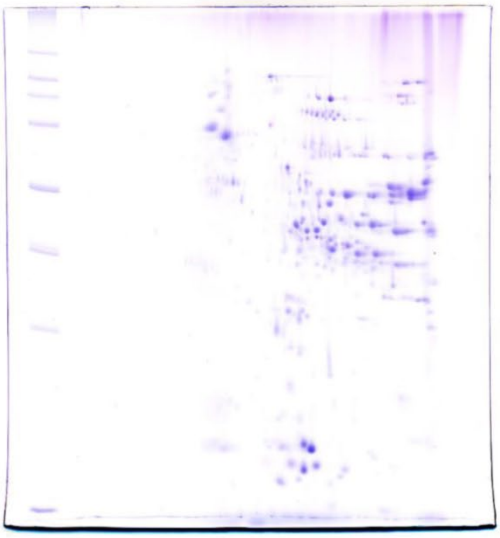

JB Asano-Early N treatment-
Replicate 5

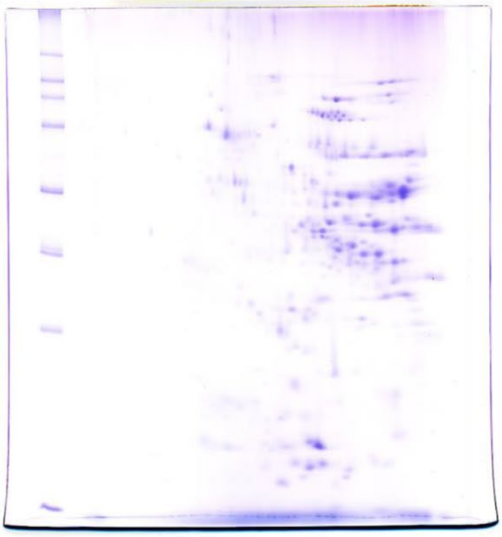

JB Asano-Split N treatment-
Replicate 5
